# The impact of interventions in the built environment on physical activity levels: a systematic umbrella review

**DOI:** 10.1186/s12966-022-01399-6

**Published:** 2022-12-20

**Authors:** Yufang Zhang, Marijke Koene, Sijmen A. Reijneveld, Jolanda Tuinstra, Manda Broekhuis, Stefan van der Spek, Cor Wagenaar

**Affiliations:** 1 Expertise Center Architecture, Urbanism and Health, Faculty of Arts, University of Groningen, Groningen, the Netherlands; 2 Department of Health Sciences, University Medical Center Groningen, University of Groningen, Groningen, the Netherlands; 3 Department of Operations, Faculty of Economics & Business, University of Groningen, Groningen, the Netherlands; 4 Department of Urbanism, Delft University of Technology, Delft, the Netherlands

**Keywords:** Urban intervention, Built environment, Physical activity, Public health, Review

## Abstract

Physical activity is good for people’s health. The relationship between the built environment and physical activity has been well documented. However, evidence is both scarce and scattered on specific urban interventions, i.e., intentional redesigns of the built environment that promote physical activity accompanied by pre- and post-effect measurement. This umbrella review aims to synthesize the findings of systematic reviews focused on these urban interventions. We followed the PRISMA 2020 and JBI umbrella review protocol guidelines and searched seven databases covering the period between Jan 2010 and April 2022 using keywords relating to the built environment, health, physical activity, and interventions. This yielded seven systematic reviews, in which we identified several urban interventions that can promote physical activity. We found positive effects of urban interventions on physical activity regarding park renovations, adding exercise equipment, introducing a (new) pocket park, improving cycling environments, improving walking & cycling environments, as well as multi-component initiatives for active travel and enhancing the availability & accessibility of destinations. The findings suggest that the urban environment can effectively promote physical activity, especially by adding various facilities and destinations and by making the environment better suitable for active use.

**Supplementary Information:**

The online version contains supplementary material available at 10.1186/s12966-022-01399-6.

## Introduction

The built environment has a significant impact on health behaviors and outcomes [1–5], particularly in urban settings. This is relevant because over half of the global population (55%) has been living in cities since 2008 [6]. Moreover, more than two-thirds (68%) of the world population is predicted to live in cities by 2050 [6]. To cope with the global urbanization trend and its challenges to human health, World Health Organization (WHO) initiated the ‘Healthy Cities’ movement, which emphasizes the importance of urban planning in improving the health and well-being of citizens [7, 8].

Physical activity (PA) is one way in which the built environment affects health [9]. In other words, a well-designed built environment has the potential to facilitate PA. Evidence has shown that a walkable environment (e.g., high density, more cross-sections, better access to facilities) can promote walking behavior [10–14] and adequate green spaces in residential areas are often associated with more PA [15]. Adequate PA has been shown to decrease risks of noncommunicable diseases such as diabetes and cardiovascular diseases [16–18].

Although there is evidence for the relationship between the built environment and PA, the effects of specific interventions that promote PA are less well studied. Most studies investigated the cross-sectional relationship between PA and built environment dimensions [3, 4, 19]. However, for the built environment, it seems more appropriate to do a pre- and post-intervention comparison, because the outcomes are then mostly impacted by the urban intervention, rather than by contextual factors such as population demographics and other characteristics of the area. The US Community Preventive Services Task Force, for example, has published a list of intervention approaches with strong or sufficient evidence regarding effectiveness to enhance PA [20]. Unfortunately, these types of longitudinal, pre- and post-intervention studies are far less available, which is thus also the case for literature reviews. Our systematic umbrella review, therefore, aims to synthesize evidence on which specific urban interventions promote PA. These insights will enable urban practitioners to create healthier urban environments.

## Methods

For this systematic umbrella review, we followed both the PRISMA 2020 guidelines (Preferred Reporting Items for Systematic Reviews and Meta-Analyses) [21] and the JBI Umbrella Review Protocol guidelines [22].

### Search strategy

A literature search was conducted to find systematic reviews that focused on the impact of urban interventions on people’s PA levels. Seven electronic databases were searched: Scopus, Web of Science (core collection), Medline, PsycINFO, EMBASE, SocIndex and Cochrane Library. The search was conducted using keywords related to ‘built environment’, ‘health’, ‘physical activity’ and ‘interventions’ (see Table 1). We searched for literature published between January 1, 2010 and April 20, 2022. The searches were conducted by two reviewers (YZ and MK) to prevent any errors.

**Table 1:** Keywords used in electronic database searches

Categories	Keywords used in the searches
Built environment	"built environment" OR "urban environment" OR "physical environment" OR "urban design" OR "urban planning" OR "public space" OR neighborhood^*^ OR neighbourhood^*^ OR "spatial" OR "town planning" OR "city planning" OR "healthy cit^*^"
	AND
Health	health OR "well-being" OR "mental fatigue" OR "depression" OR "stress" OR "burn-out" OR "obesity" OR "overweight" OR "physical endurance" OR "cardiorespiratory fitness" OR "physical fitness"
	AND
Physical activity	"physical activity" OR "physically active" OR "exercise" OR "walk^*^" OR "pedestrian^*^" OR "^*^cycling" OR "biking" OR "active travel*" OR "active transport^*^" OR "active commut^*^" OR "active play^*^" OR "recreation" OR "leisure" OR "sport^*^" OR "play^*^" OR sedentary
	AND
Interventions	intervention OR design OR garden OR (park NOT parking) OR green OR "urban development" OR "urban expansion" OR "cyclability" OR "walkability" OR "pedestrianization" OR "pedestrianisation" OR "urban renewal" OR natur^*^ OR "forest" OR "blue space" OR "playground" OR "infrastructure"

^*^The Asterisk (*) allows keywords to be searched in different versions of the word

### Study selection

Review papers were selected if they met the following eligibility criteria:

1. the paper was a systematic review;
2. the paper reviewed interventions in the (public space of the) urban built environment to promote PA;
3. the outcomes were measured after a specific intervention (or interventions) was implemented, meaning that the outcomes were based on pre- and post-intervention analysis; the primary outcomes were PA levels.

There were no restrictions regarding language.

**Fig. 1:**
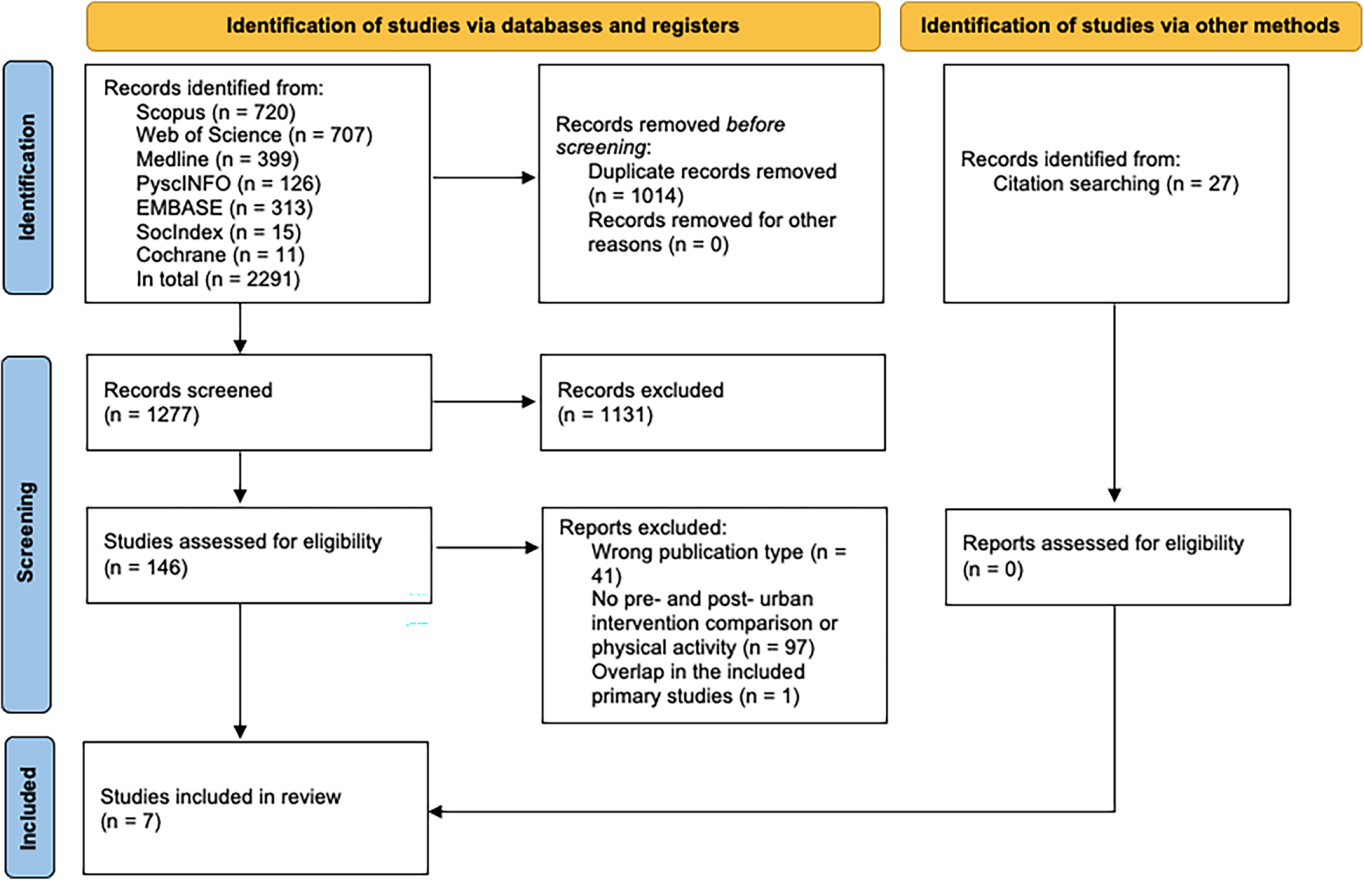
Selection procedure, based on Flowchart PRISMA

After the duplicates were removed, two reviewers (YZ & MK) independently screened the paper titles according to the eligibility criteria. Any disagreements were resolved between the two reviewers. A third reviewer (SvdS) was available if the two reviewers could not resolve any disagreements; this did not occur. The same procedure was followed for screening abstracts and full papers. The entire selection procedure is shown in Fig. 1. Because this is an umbrella review, or a review of reviews, it is possible that the included reviews overlap in terms of included studies. To prevent overlap and overrepresentation of primary studies, reviews that met the eligibility criteria could be excluded if their included primary studies were too similar to those of another review. In this case, the review that showed the most overlap was excluded. The reference lists of the included papers were searched for additional papers that met the eligibility criteria. No additional papers were found after title and abstract screening.

### Data extraction and synthesis

The study characteristics were extracted by reviewer one (YZ) and checked by reviewer two (MK) (see Table 2). The study designs employed among the studies in each included review were summarized. Controlled quasi-experiments are studies that adopted a pre-post assessment design with a comparison group. Uncontrolled quasi-experiments are studies that adopted a pre-post assessment design without a comparison group. Studies that conducted repeated observations of the same group over time are referred to as longitudinal cohort studies. Tables 4 and 5, explaining the interventions and showing the main results, were developed by both reviewers.

**Table 2:** Characteristics of the included reviews

	**Author & publication year**	**Number of included studies**	**Included studies (range in years)**	**Age groups**	**Location**	**Review type**	**Study design**	**Study objectives**	**Interventions**	**Key findings**
1 [23]	Audrey & Batista-Ferrer (2015)	32	1991–2014	Children and young people	North America, Oceania, and Europe	Systematic review	quasi-experiments – controlled (81%); quasi-experiments – uncontrolled (19%)	To examine evidence from interventions in the urban environment in relation to health behaviors and physical and mental health outcomes of children and young people	Park and playground modifications, road traffic safety measures, multi-component community-based interventions and promoting active travel	Some evidence was found for interventions to reduce road traffic injuries, to increase active travel, in relation to a multi-component health initiative. Limited evidence was identified for interventions to increase park and playground usage
2 [24]	Hunter, et al. (2019)	38	2002–2016	All age groups	North America, Oceania, and Europe	Systematic review	quasi-experiments – controlled (89%); quasi-experiments – uncontrolled (11%)	To review and synthesize the evidence of urban green space (UGS) interventions; to discuss the findings at an expert review panel; and to develop recommendations on UGS interventions to policymakers, practitioners and researchers	A physical change to green space in urban areas, including improving UGS or developing new UGS, or a combination of physical change to UGS supplemented by a specific UGS-usage promotion program	The use of certain UGS interventions was found supportive for health, social and environmental benefits, in particular park-based and greenway/trail-based interventions employing a dual approach. In other UGS interventions inconclusive finding was found
3 [25]	Kärmeniemi, et al. (2018)	51	2003–2015	All age groups	North America, Oceania, Europe, and Asia	Systematic review	quasi-experiments – controlled (61%); longitudinal cohort studies (39%)	To identify the determinants of the built environment that are related to physical activity (PA) and to evaluate the effects of built environment changes on PA	Objective built environment measures (such as new infrastructure and park and playground improvements) and perceived built environment measures (such as safety and aesthetics)	Built environment changes can lead to PA change. For example, higher accessibility and new infrastructure for walking, cycling and public transportation are related with higher PA levels
4 [26]	Panter, et al. (2019)	13	1987–2015	All age groups	North America, Oceania, Europe, and Asia	Systematic review	quasi-experiments – controlled (46%); quasi-experiments – uncontrolled (54%)	To understand how built environment interventions impact PA and walking and cycling behavior and to understand their effectiveness of lack thereof	Interventions to promote walking and cycling: accessibility and connectivity, traffic and personal safety and quality/experience	Three intervention categories were identified that promote walking and cycling: improving accessibility and connectivity, improving traffic and personal safety, and improving walking and cycling experience
5 [27]	Smith, et al. (2017)	28	1979–2015	All age groups	North America, Oceania, and Europe	Systematic review	not reported	To identify which built environment interventions are effective in promoting PA in local residents and to build on the evidence base by considering intervention cost and the differential effects of different demographics	Active transport infrastructure, parks and playgrounds, walkability components	Potential ways to promote activity in children and adults were found: neighborhood walkability improvement, parks and playgrounds’ quality enhancement, and adequate active transport infrastructure provision
6 [28]	Stappers, et al. (2018)	19	2005–2017	All age groups	North America, Oceania, and Europe	Systematic review	quasi-experiments – controlled (89%); quasi-experiments – uncontrolled (11%)	To update and specify the evidence on the effects of different types of infrastructural interventions on PA, active travel and sedentary behavior in adults	On- and off-road bicycle and/or walking trails, built environment infrastructural changes	The impact of built environment infrastructural changes varies a lot across types of intervention and outcome measure. Infrastructural interventions are not always effective in promoting PA or active travel
7 [29]	Tcymbal, et al. (2020)	36	2007–2020	All age groups	North America, Oceania, Europe, and Asia	Systematic review	quasi-experiments – controlled and uncontrolled (69%); longitudinal cohort studies (31%)	To identify built environment determinants of PA and thereby taking gender into account	Interventions related to transport, recreation and household	Creating a new infrastructure for walking, cycling, and public transportation showed a positive effect on PA. Improving walking and cycling infrastructure had no effect on the overall PA, but it had a positive effect on active transportation

First, an overview was made of all the specific changes in the built environment (later referred to as ‘BE changes’) that met the aim and eligibility criteria of this systematic umbrella review. The identified BE changes were then categorized in 16 interventions, in three intervention categories, along with their PA outcomes.

Based on the available data, three types of changes in PA were chosen for data synthesis: usage (the difference in how often a place is used or visited), combined PA (this includes PA, moderate to vigorous PA and leisure time PA) and active travel (which means being physically active, often walking or cycling, to a specific destination).

The PA outcomes are presented as: positive (↑), negative (↓) and null (0). Positive means an effect in the expected direction (in this case promoting PA), negative means an effect contrary to the expected direction, and null means that the intervention showed no effect in the expected direction. When interventions show a mixed result, with more than one possible outcome, we deem it as promising if the percentage of positive outcomes is 60% or higher.

### Quality assessment

The quality of the included review papers was assessed according to the JBI Critical Appraisal Checklist for Systematic Reviews and Research Syntheses [30]. Two reviewers (YZ & MK) conducted the assessment individually. Any disagreements were discussed until consensus. The JBI checklist contains 11 assessment items. Reviews that included zero to four assessment items were considered low quality, five to seven as moderate, and eight or above as high quality.

## Results

In total, 2291 review papers were identified in the database search, of which 1277 remained after duplicates were removed. After reviewing titles and abstracts, 146 papers remained for fulltext review (see Fig. 1). This led to the inclusion of eight papers [23–29, 31]. Two of the eight papers were written by the same first author and showed much overlap in included studies and results [24, 31]. The paper with the most overlap in primary studies was excluded [31]. This resulted in the inclusion of seven systematic review papers (Table 2). The quality assessment showed that three of the seven reviews have high methodological quality and four have a moderate quality (Table 3). Although some methodological limitations were found, none of the included reviews had low quality. Therefore, our quality assessment excluded no reviews.

**Table 3:** Quality scores of the included systematic reviews

	**Author**	**Quality assessment**											Quality^*^
		Is the review question clearly and explicitly stated?	Were the inclusion criteria appropriate for the review question?	Was the search strategy appropriate	Were the sources and resources used to search for studies adequate?	Were the criteria for appraising studies appropriate?	Was critical appraisal conducted by two or more reviewers independently?	Were there methods to minimize errors in data extraction?	Were the methods used to combine studies appropriate?	Was the likelihood of publication bias assessed?	Were recommend-dations for policy and/or practice supported by the reported data?	Were the specific directives for new research appropriate?	
1 [23]	Audrey & Batista-Ferrer (2015)	yes	yes	yes	yes	unclear	no	yes	unclear	yes	yes	yes	high
2 [24]	Hunter, et al. (2019)	yes	yes	yes	yes	yes	yes	yes	yes	yes	yes	yes	high
3 [25]	Kärmeniemi, et al. (2018)	yes	yes	yes	yes	no	n/a	yes	yes	yes	unclear	yes	moderate
4 [26]	Panter, et al. (2019)	yes	unclear	yes	yes	unclear	yes	yes	yes	no	no	yes	moderate
5 [27]	Smith, et al. (2017)	yes	yes	yes	yes	yes	no	unclear	yes	unclear	no	yes	moderate
6 [28]	Stappers, et al. (2018)	yes	yes	yes	yes	no	n/a	no	no	yes	no	yes	moderate
7 [29]	Tcymbal, et al. (2020)	yes	yes	yes	yes	yes	yes	yes	yes	yes	yes	yes	high

^*^0–4 times yes equals low quality; 5–7 times yes equals moderate quality; 8–11 times yes equals high quality

### Study characteristics

Table 2 shows the study characteristics of the seven included systematic reviews. The reviews were published between 2015 and 2020. In total, the articles covered 217 primary studies, published between 1979 and 2020. All primary studies were published after the year 2000 except for one that was published in 1979. Quasi-experimental designs were the most often used in the primary studies (Table 2). One article focused on children and young people, whereas the other articles did not focus on a specific age group. All articles included studies from high-income countries, mostly from North America, Oceania, and Europe. However, Asia was also covered in two articles [25, 29].

### The impact of urban intervention on PA

Data extraction and synthesis produced three intervention categories and 16 corresponding interventions. The three intervention categories are: 1) park and playground interventions, 2) interventions aimed at walking and cycling, and 3) community-based interventions. The first category includes interventions to improve the facilities or environment of parks and/or playgrounds. The second category includes interventions to improve opportunities for walking and cycling by increasing the availability, accessibility, and safety of routes. The last category regards interventions taking place in a community (or neighborhood) setting. These are often broader and address different aspects of the area. Table 4 explains the interventions. For each intervention, examples are given of the specific BE changes that were implemented in the different studies.

**Table 4:** Intervention categories and corresponding interventions

Interventions	Examples for specific BE changes for each intervention
**Park and playground interventions**	
1. Park renovations	Upgrading paths, adjusting seating areas, adding walking trails, greenery, barbecue or picnic areas, equipment, lighting, and a fenced leash-free area for dogs
2. Playground renovations	Installing new components (play equipment, seating, additional safety surfacing, and waste facilities), removing existing components
3. Park & playground renovations	A combination of interventions 1 and 2
4. Exercise equipment	Introducing outdoor exercise equipment/fitness equipment/family fitness zones
5. Introducing a new (pocket) park	Redesigning existing green spaces into pocket parks to increase seating areas and walking trails
6. Multi-component green initiatives	Replacing vacant land with new public park, redesigning existing parks, landscaping, planting flower bulbs in front yards, constructing wall gardens, greening streets, adding a greenway
**Interventions aimed at walking and cycling**	
7. Improving walking environments	Adding new sidewalks, walking paths, crosswalks, pedestrian crossing signs/signals, median islands, four-way stops, safe routes to schools, in-pavement crosswalk lighting. Renovating/repairing walking paths, repainting crosswalk lines, filling gaps in the sidewalk network
8. Improving cycling environments	Installing cycle lanes, e.g., striped cycle lanes, separated bicycle paths, on and off-street/road bike lanes and new cycle lanes/trails. Adding bicycle boulevards, providing cycling-related facilities (bike storage/parking), improving cycling traffic (signs and crossings)
9. Improving walking & cycling environments	Improving crosswalks, sidewalks, bike parking, installing traffic calming features (raised platforms, zebra crossings) and parking bays, creating safe places to walk. Adding off-road paved paths, adding new walking and cycling routes
10. Greenway & trail	Adding/extending a greenway, introducing/extending a trail, renovating/extending a railway to a multi-use trail
11. Traffic free bridge	Adding new pedestrian and cycle bridges
12. Improving infrastructure system	A combination of adding routes for walking and cycling, rail-infrastructure, bike parking, street improvements. A combination of adding a new avenue, parking lots, on-road walking and cycling road. Construction of an off-road guideway for buses, with a parallel path for walking and cycling
13. Multi-component initiatives for active travel	Wayfinding, improving crossings, sidewalks and from/to school environments, traffic calming measures, altered drop off/pick-up zones, creating parking bays, cycle lanes and pedestrian overpasses. Adding painted crosswalks, introducing and improving signage, parks, and bike racks, extending a walking path in conjunction with a subway expansion project
**Community-based interventions**	
14. Increase density	Population density, service/job density, recreation facility density and residential density
15. Availability & accessibility of destinations	Increase number and accessibility of destinations, land-use mix, public transit availability/accessibility, sport facility availability, reduce distance to parks
16. Street network initiatives	Changes in street connectivity and road characteristics

Table 5 shows the PA outcomes for each intervention. When all the results are taken together, the table includes 274 BE changes. Positive effects were reported for 149 (54.4%) of these BE changes, null effects for 112 (40.9%) and negative effects for 13 (4.8%) of the BE changes. The outcomes for each of the three intervention categories are presented separately in the following subsections.

**Table 5:** Results for the effects of urban interventions on physical activity, as positive (↑), null (0) or negative (↓)

		**Usage**		**Combined PA**		**Active travel**		**Total amount & %**
**Park and playground interventions**	1) Park renovations	↑	[1]^*^2; [5]^*^3; [7]^*^4	↑	[5]^*^5; [7]^*^2	↑		↑ 16 (66.7%)
		0	[2]^*^1	0	[5]^*^3; [7]^*^1	0		0 5 (20.8%)
		↓	[1]^*^1; [5]^*^1	↓	[7]^*^1	↓		↓ 3 (12.5%)
	2) Playground renovations	↑		↑		↑		↑
		0		0	[1]^*^1; [2]^*^1	0		0 2 (100%)
		↓		↓		↓		↓
	3) Park & playground renovations	↑	[2]^*^2	↑	[2]^*^1; [3]^*^6	↑		↑ 9 (50%)
		0	[1]^*^1 [2];^*^2	0	[1]^*^2; [2]^*^2; [3]^*^2	0		0 9 (50%)
		↓		↓		↓		↓
	4) Exercise equipment	↑		↑	[5]^*^2; [7]^*^2	↑	[5]*1	↑ 5 (62.5%)
		0	[2]^*^1	0		0	[5]*1	0 2 (25%)
		↓	[1]^*^1	↓		↓		↓ 1 (12.5%)
	5) Introducing a new (pocket) park	↑	[5]^*^1; [7]^*^2	↑	[5]^*^1; [7]^*^3	↑		↑ 7 (87.5%)
		0	[2]^*^1	0		0		0 1 (12.5%)
		↓		↓		↓		↓
	6) Multi-component green initiatives	↑		↑		↑		↑
		0		0	[2]^*^2	0		0 2 (100%)
		↓		↓		↓		↓
**Interventions aimed at walking and cycling**	7) Improving walking environments	↑		↑		↑	[1]^*^3	↑ 3 (50%)
		0		0	[1]^*^1; [4]^*^1	0	[1]^*^1	0 3 (50%)
		↓		↓		↓		↓
	8) Improving cycling environments	↑	[5]^*^3	↑	[4]^*^4; [6]^*^3	↑	[1]^*^1	↑ 11 (61.1%)
		0		0	[4]^*^1; [5]^*^1 [6]^*^3	0	[1]^*^1	0 6 (33.3%)
		↓		↓		↓	[5]^*^1	↓ 1 (5.6%)
	9) Improving walking & cycling environments	↑	[5]^*^2; [7]^*^3	↑	[4]^*^1; [5]^*^3 [6]^*^1; [7]^*^5	↑	[5]^*^3	↑ 18 (69.2%)
		0		0	[7]^*^5	0	[5]^*^1	0 6 (23.1%)
		↓		↓	[7]^*^2	↓		↓ 2 (7.7%)
	10) Greenway & trail	↑		↑	[4]^*^1; [5]^*^2; [6]^*^2	↑	[5]^*^1	↑ 6 (31.6%)
		0		0	[2]^*^3; [4]^*^3; [5]^*^1 [6]^*^2	0	[1]^*^1; [2]^*^2; [5]^*^1	0 13 (68.4%)
		↓		↓		↓		↓
	11) Traffic free bridge	↑	[4]^*^1	↑	[5]^*^1	↑	[5]^*^1	↑ 3 (50%)
		0		0	[6]^*^3	0		0 3 (50%)
		↓		↓		↓		↓
	12) Improving infrastructure system	↑		↑	[3]*11; [6]^*^1	↑	[3]^*^6	↑ 18 (50%)
		0		0	[3]^*^9; [6]^*^3	0	[3]^*^4	0 16 (44.4%)
		↓		↓	[3]^*^2	↓		↓ 2 (5.6%)
	13) Multi-component initiatives for active travel	↑		↑	[1]^*^1; [7]^*^1	↑	[1]^*^4	↑ 6 (66.7%)
		0	[5]^*^1	0	[7]^*^1	0	[1]^*^1	0 3 (33.3%)
		↓		↓		↓		↓
**Community-based interventions**	14) Increase density	↑		↑	[3]^*^2; [5]^*^1	↑	[3]^*^3; [5]^*^1	↑ 7 (38.9%)
		0		0	[3]^*^7	0	[3]^*^3; [5]^*^1	0 11 (61.1%)
		↓		↓		↓		↓
	15) Availability & accessibility of destinations	↑		↑	[3]^*^7; [5]^*^2; [7]^*^7	↑	[3]^*^8; [5]^*^4	↑ 28 (62.2%)
		0		0	[3]^*^9; [7]^*^4	0	[3]^*^3	0 16 (35.6%)
		↓		↓	[3]^*^1	↓		↓ 1 (2.2%)
	16) Street network initiatives	↑		↑	[3]^*^5; [5]^*^1; [7]^*^3	↑	[3]^*^2; [5]^*^1	↑ 12 (41.4%)
		0		0	[3]^*^8; [7]^*^3	0	[3]^*^3	0 14 (48.3%)
		↓		↓	[3]^*^2; [7]^*^1	↓		↓ 3 (10.3%)

[x] refers to the article number as used in Table 2

^*^x shows the number of BE changes in that article

↑: positive effect; 0: null effect; ↓: negative effect

Total amount & %: the total amount is the summary of all the BE changes in that row, % shows the percentage of ↑/0/↓ for that intervention

Combined PA includes physical activity, moderate to vigorous physical activity and leisure time physical activity

#### Park and playground interventions

Five of the seven included systematic reviews reported on interventions in parks and/or playgrounds [23–25, 27, 29]. In total, this category includes 62 park and/or playground related BE changes. Most BE changes showed either a positive (59.7%) or a null (33.9%) effect in terms of usage or combined PA. Only 6.5% showed a negative effect. Most of the articles reported on usage or combined PA for this category; only one article included two BE changes that were measured for active travel [27].

Introducing a new (pocket) park, park renovations and introducing exercise equipment are the three most promising subcategories in terms of promoting PA, as shown in the last column of Table 5. First, the introduction of a new (pocket) park included eight BE changes, of which seven (87.5%) showed a positive and one (12.5%) a null effect. Second, park renovations included 24 BE changes, of which 16 (66.7%) showed a positive effect, five (20.8%) a null effect and three (12.5%) a negative effect. Finally, adding exercise equipment included eight BE changes, of which five (62.5%) showed a positive, two a null (25%) and one (12.5%) a negative effect.

#### Interventions aimed at walking and cycling

All seven included systematic reviews reported on interventions aimed at walking and cycling [23–29]. In total, this category includes 120 BE changes. Most BE changes showed either a positive (54.2%) or a null (41.7%) effect in terms of usage or combined PA. Only 4.2% showed a negative effect. Most articles reported on combined PA or active travel, which is logical considering that the BE changes concern walking, cycling and traffic in a broader sense.

Cycling, walking & cycling and multi-component initiatives for active travel are the three subcategories that show the most promise in terms of promoting PA. Of 18 BE changes to promote cycling, 11 (61.1%) showed a positive, six (33.3%) a null and one (5.6%) a negative effect. Of 26 BE changes to promote both walking & cycling, 18 (69.2%) showed a positive, six (23.1%) a null and two (7.7%) a negative effect. Finally, the multi-component initiatives for active travel contains nine BE changes, of which six (66.7%) showed a positive effect and three (33.3%) a negative effect.

#### Community-based interventions

Three of the seven included systematic reviews reported on community-based initiatives [25, 27, 29]. In total, this category includes 92 community-based BE changes. Most BE changes showed either a positive (51.1%) or a null (44.6%) effect in terms of usage or combined PA. Only 4.4% of the BE changes showed a negative effect. All interventions were measured either for combined PA or active travel. There were no outcomes for usage in this category.

Enhancing the availability & accessibility of destinations is most promising in terms of promoting PA, containing 45 BE changes, of which 28 (62.2%) showed a positive, 16 (35.6%) a null and one (2.2%) showed a negative effect.

### Methodological limitations of the included reviews

We observed several methodological limitations in the included reviews. First, only three of the seven reviews clearly stated that their quality assessment was conducted by at least two reviewers independently (Table 3), indicating a relatively high risk of a low-quality assessment. Second, five of the included reviews applied extra methods to minimize data extraction errors. Third, the authors used different definitions of urban interventions and PA across the included reviews. PA, for example, was measured for different outcomes in terms of overall PA, moderate to vigorous PA, walking, cycling behaviors and steps per day. PA was also measured differently in such ways as duration, frequency, or intensity across reviews. Differences were also found in urban interventions. For example, extending existing greenways and adding entirely new greenways were both categorized as greenway interventions (Table 4 presents more examples). Fourth, longitudinal studies used very different periods of exposure to the interventions. In one included review, the exposure period ranged from one month to 15 years [25]. Finally, some of the included reviews [26, 29] combined the findings for children, young people, and adults without differentiating between age groups.

## Discussion

### Main findings

To our knowledge, this is the first systematic umbrella review on the relationship between specific urban interventions in the built environment and PA. We identified 16 interventions (divided into three categories) of which seven were promising: park renovations, adding exercise equipment, introducing a new (pocket) park, improving cycling environments, improving both walking & cycling environments, multi-component initiatives for active travel, and enhancing availability & accessibility of destinations. Each of the three intervention categories, namely park and playground interventions, interventions aimed at walking and cycling and community-based interventions, showed at least one promising intervention. Therefore, we conclude that all three intervention categories have the potential to contribute to the promotion of PA.

In the category of park and playground interventions, renovation of parks (e.g., upgrading paths, adjusting seating areas, adding barbecue or picnic areas, etc.) was positively related to PA. Although playground renovation was found less effective, this intervention was reported by only two primary studies. The combination of park & playground renovations showed less impact than park renovations alone. This might be explained by the fact that playgrounds are mostly used by children, and two of the three reviews that reported on park & playground renovations made no distinction between age groups. This combination of renovations might therefore show a less positive effect, because it has less impact on older age groups. Other interventions that can promote PA include adding exercise equipment (e.g., fitness equipment and family fitness zones) and a new (pocket) park. It can therefore be concluded that introducing new destinations or facilities is beneficial for promoting PA. Multi-component green initiatives were found to have no effect on PA. However, the outcome of this intervention was based on only one of the included reviews, and further evidence is required to fully evaluate the effectiveness of this intervention.

In the second intervention category, interventions aimed at walking and cycling, the intervention improving solely the walking environment showed mixed results with half of the BE changes showing a positive effect and half a null effect. In contrast, interventions addressing a combination of BE changes were more promising to promote PA, being multi-component initiatives for active travel and improving walking & cycling environments. Therefore, we argue that in this case it is more promising to promote PA with combined interventions. Strikingly, however, interventions focusing solely on improving the cycling environment also show promise to improve PA. We thus learn that more comprehensive interventions are mostly more effective with the difference in effectiveness between walking and cycling interventions requiring further study.

In the community-based intervention category, only interventions enhancing availability & accessibility of destinations had a positive impact on PA. This finding is in line with a previous umbrella review, which found enhancing overall access to facilities and access to public transport to have positive effects on PA [32]. Enhancing density, which has been recognized as an important indicator for PA [33–35], however, showed fewer positive effects in our results. This difference in outcome might be caused by different measuring methods. Increasing density alone (e.g., housing density) might not lead to an increase in PA [36], but often when an area gains density, other functions or destinations are also added or increased, and those do have a positive effect on PA. It can therefore be argued that density itself does not have actual impact on PA, but a positive impact can be shown when increased density is combined with more diverse land use or more access to varied destinations. Finally, street network initiatives showed mixed, mostly null, outcomes, which contradicts findings from other studies where it was found to have a positive effect [32, 33]. This contradiction might be explained because street network initiatives is a very broad term that can include many different interventions, meaning that different studies may have investigated different initiatives under the same name.

The findings from this systematic umbrella review showed that all three intervention categories included interventions that can promote people’s PA levels. Even so, it remains difficult to explain why some interventions work and others do not, especially when interventions seem comparable, such as park renovations and park & playground renovations or improving walking environments and improving cycling environments. The insufficiency of evidence on the effectiveness of intervention for increasing PA is also reported in the Guide to Community Preventive Services [37]. This points out that designing urban interventions to change people’s behavior is very challenging and the same is true for researching those interventions [38]. Certain interventions may be effective in a certain context or environment but might not work in other instances.

### Strengths and limitations

The main strength of our systematic umbrella review is that we focused on studies that measured PA levels before and after intervention, providing relatively strong evidence for a causal relationship between the urban intervention and PA. Most previous umbrella reviews on this topic include cross-sectional studies, limiting the potential for inferences on causal relationships [32]. Our systematic approach and umbrella review strategy led to a comprehensive overview of the evidence. The included studies were all moderate and high quality, which led to reliable outcomes.

Our review has potential limitations. First, some methodological limitations were identified among the included reviews, such as low-quality primary studies that lacked methods to minimize errors. In this umbrella review, however, we could not further adjust for this within the included reviews. Nonetheless, our quality assessment showed an overall moderate-to-high quality of included reviews. Second, we synthesized evidence from studies worldwide, without differentiating for local context. Our findings may thus have been affected by the heterogeneity of settings. Furthermore, the measuring standard and the definitions for both interventions and PA varied across the studies, which might have impacted our findings. Some included reviews showed only if an intervention had an effect in the expected direction (i.e., a positive or a null outcome), not whether it had a negative effect [24], while other reviews did. This may have impacted the null and negative outcomes slightly but should have no influence on the positive outcomes. Finally, our findings are based on all age groups combined even though the impact of some interventions might vary across ages. However, we cannot report on evidence per age group, as not enough reviews reported on this. 

### Implications for practice and policy

As we focused on specific urban interventions, the three intervention categories identified in our paper can be directly useful for practitioners and policymakers in the planning of urban interventions to create health-promoting environments. However, urban interventions are context-related and often multi-interpretable due to the variations in their definition which may help to explain inconsistencies in the evidence. This has implications for the contribution of robust scientific evidence to date on practices and policies to inform health-promoting environments. There is, however, a growing body of knowledge on how urban interventions are associated with PA which should be considered by urban design practitioners.

### Implications for future research

We found some conflicting findings, possibly due to methodological limitations of the included reviews, such as combining geographical contexts and age groups and the limited quality of some of the primary studies. This implies that to gather knowledge on what works where, future research findings should be differentiated for context, e.g., for demographics and environmental characteristics of an area or region. There is no ‘one size fits all’ approach; interventions need to be tailored based on local contexts and population needs.

In addition, our findings on street network initiatives conflicted with previous studies, possibly due to differing definitions. This shows the need to standardize terminology; consistent definitions and data synthesizing of ‘interventions’ and ‘physical activity’ is required for further research. Without standardization, the interpretation of the findings cannot offer strong support to causal relationships between PA and BE. We found very little evidence based on low-income countries, and it is well documented that low-income countries have higher rates of disease resulting from inactive lifestyles [39]. More research on low-income countries is needed. More longitudinal research with a pre- and post-measurement is needed to provide a better understanding of the causal relationship between urban environments (or the built environment in general) and population PA. There is a need for more high-quality studies to provide more conclusive evidence.

## Conclusion

Our findings show that three urban intervention categories (park and playground interventions, interventions aimed at walking and cycling, and community-based interventions) have the potential to promote PA. However, there is a need for standardized definitions and research methods which will help reduce the gap between scientific research and practice and would better contribute to policies aimed to design healthier cities.

## Supplementary Information

**Additional file 1.**

## Data Availability

Not applicable.

## Abbreviations

BE: Built environment
PA: Physical activity
UGS: Urban green spaces
